# Complete Genome Sequence of *Streptomyces* Phage Spernnie

**DOI:** 10.1128/MRA.01344-20

**Published:** 2021-01-07

**Authors:** Nathaniel Tate, Loraine Melbern, James Clark, Isla Hernandez, Mei Liu, Ben Burrowes

**Affiliations:** a Department of Biochemistry and Biophysics, Texas A&M University, College Station, Texas, USA; b Center for Phage Technology, Texas A&M University, College Station, Texas, USA; Loyola University Chicago

## Abstract

*Streptomyces* spp. are Gram-positive soil bacteria that have been reported in some cases to cause acute and chronic infections, including mycetomas, pneumonia, and septicemia. Here, we present *Streptomyces* sp. strain Mg1 phage Spernnie. Spernnie is a temperate siphophage containing 89 predicted coding genes in a 50,834-bp genome sequence.

## ANNOUNCEMENT

*Streptomyces* sp. strain Mg1, like all other streptomycetes, is a Gram-positive saprotrophic soil bacterium. *Streptomyces* strains are capable of causing infections in immunocompromised patients, the most common clinical finding being chronic skin infections known as mycetomas (1). Although much rarer, cases of pneumonia and septicemia have been reported due to *Streptomyces* infection (1). *Streptomyces* sp. Mg1 is capable of producing the antibiotic chalcomycin A (2).

Bacteriophage Spernnie was isolated from a soil sample collected in Torrey, NY, in August 2019 using the double-agar overlay technique (3) with *Streptomyces* sp. Mg1 as the host strain (4), grown at 30°C on nutrient agar supplemented with 10 mM MgCl_2_, 8 mM Ca(NO_3_)_2_, and 0.5% glucose. Virion morphology was determined by negative staining of samples with 2% (wt/vol) uranyl acetate and visualization by transmission electron microscopy (TEM) at the Texas A&M Microscopy and Imaging Center. Phage genomic DNA (gDNA) was purified using the modified Wizard DNA clean-up kit as described by Summer (5), prepared as Illumina TruSeq libraries using a TruSeq Nano kit, and sequenced on an Illumina iSeq 100 platform using paired-end 2 × 300-bp reads. Quality control was performed on a total of 472,632 sequence reads with FastQC (www.bioinformatics.babraham.ac.uk/projects/fastqc), and reads were manually trimmed using FastX v0.0.14 (http://hannonlab.cshl.edu/fastx_toolkit/download.html). The genome sequence was assembled using SPAdes v3.5.0 with default parameters (6), which provided a single contig with 116.9-fold coverage. Sanger sequencing of a PCR product amplified off the contig ends (forward, 5′-ACCTTCCCACTGGTGTCT-3′; reverse, 5′-GAGAACCACTTCGCGTTCTAC-3′) was performed to close the genome. PhageTerm analysis was run but was unable to predict the genome termini (7). Structural annotation was performed with GLIMMER v3 (8) and MetaGeneAnnotator v1.0 (9). Possible tRNAs were detected with ARAGORN v2.36 (10). Gene function was predicted using InterProScan v5.33 (11) and BLAST v2.9.0 (12). TMHMM v2.0 was used to predict any transmembrane domains (13). BLAST analysis referred to the NCBI nonredundant database as well as the Swiss-Prot and TrEMBL (14) databases. progressiveMauve v2.4 was used to calculate genome-wide nucleotide sequence similarity (15). All annotations were done using annotation tools in the Galaxy and Web Apollo instances hosted by the Center for Phage Technology at https://cpt.tamu.edu/galaxy-pub (16–18). Unless otherwise specified, all software was used with default parameters.

The Spernnie genome sequence is 50,834 bp long, with a GC content of 65.7%. The coding density is 92.3%, and the genome contains 89 putative protein coding genes, 38 of which were assigned putative functions. Sequence analysis suggests that Spernnie is a siphovirus, which was confirmed visually by TEM. Due to the putative finding of characteristic temperate phage genes such as an integrase and immunity region, Spernnie appears to be a temperate siphophage. Spernnie’s genome contains a lysis cassette with a predicted *N*-acetylmuramidase endolysin as well as a putative embedded holin/antiholin pair. Spernnie is related to phages in the *Arquatrovirinae* subfamily, the most closely related being *Streptomyces* phage Caelum (GenBank accession number MK524524), with 67.3% nucleotide sequence similarity.

### Data availability.

The genome of Spernnie is deposited in GenBank under accession number MT701594.1. The associated BioProject, SRA, and BioSample accession numbers are PRJNA222858, SRR11558338, and SAMN14609634, respectively.
